# The Extraordinary Nature of Illusion

**DOI:** 10.3201/eid1411.000000

**Published:** 2008-11

**Authors:** Polyxeni Potter

**Affiliations:** Centers for Disease Control and Prevention, Atlanta, Georgia, USA

**Keywords:** Art science connection, emerging infectious diseases, art and medicine, Giuseppe Arcimboldi, Arcimboldo, Vertumnus, mannerism, emerging infections, Chimera, surrealism, about the cover

**Figure:**
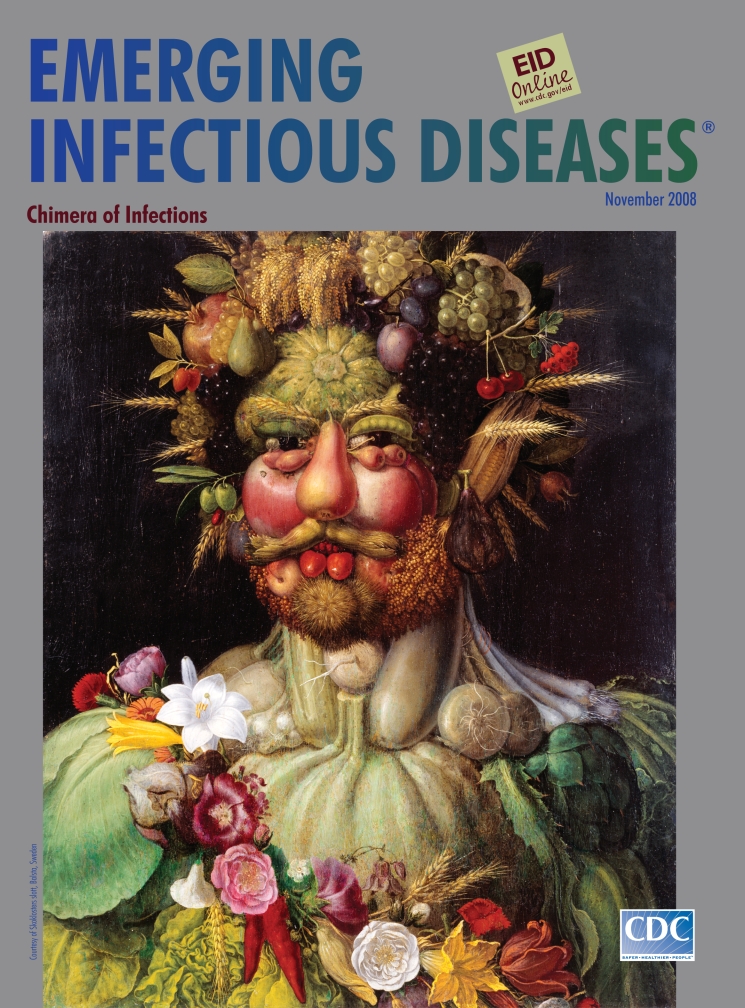
**Giuseppe Arcimboldi or Arcimboldo (1527–1593)** **Vertumnus (1590–91)** Oil on wood (71 cm × 57.5 cm) Skoklosters slott, Balsta, Sweden

“When our first encounter with some object surprises us and we find it novel or very different from what we formerly knew or from what we supposed it ought to be, this causes us to wonder and be astonished at it,” wrote 17th-century philosopher René Descartes in his Passions of the Soul. Indeed, astonishment awaits anyone who views for the first time the work of Giuseppe Arcimboldi, Milanese painter extraordinaire, portraitist of emperors, and master of illusion (*1*).

Arcimboldi grew up in a distinguished family. He associated with philosophers and other scholars and knew the son of Bernardino Luini, a student of Leonardo, who had notes and sketchbooks given to him when the master left Milan, site of most of his experiments (*2*). In writing about the family, historian Paolo Morigia described Giuseppe as “a trustworthy gentleman with an impeccable lifestyle,” who started his artistic career at age 22 designing tapestry and stained glass with his father, also an artist (*3*). This early work at Milan Cathedral already contained elements found in his unique later style.

In the beginning of the 16th century, partly because of a plague epidemic, Milan’s position as leader in the arts was declining. Nonetheless, Arcimboldi’s reputation was strong. “This is a painter with a rare talent, who is also extremely knowledgeable in other disciplines,” wrote Morigia about Arcimboldi’s acceptance of the invitation of Emperor Ferdinand I to go to Prague. “And having proved his worth as an artist and as a bizarre painter, not only in his own country but also abroad, he has been given the highest praise” (*3*).

Arcimboldi flourished during his tenure with Ferdinand. “He was liked and treated well and received with great kindness, and the Emperor gave him a good salary worthy of his merits” (*3*). He came to know the works of such greats as Hieronymus Bosch, Peter Bruegel the Elder, and Albrecht Altdorfer. He painted portraits of the imperial family and the first series of his Four Seasons, composite heads with allegorical meanings. His work continued for succeeding Holy Roman Emperors Maximilian II and Rudolph II. In their courts, “This noble and inspired man fashioned a great number of rare and delicate works of art which caused considerable amazement” (*3*).

Apart from painting, Arcimboldi had other duties. He was designer of costumes, masques, and disguises worn during festivals by impersonators of ancient gods, the liberal arts, or anything else living or mythical. He served as architect, stage designer, engineer, and advisor to Maximilian, who delighted in animals and believed in the healing power of plants. He started a museum to house rare specimens and artifacts from as far away as the newly discovered continent of America. Later, under Rudolph, this museum would become the Art and Wonder Chambers. Arcimboldi traveled to Germany to buy art objects and exotic birds for it, which he studied from nature and incorporated in his works.

Rudolph was interested in all disciplines, from mathematics to gardening. Many scholars and artists, among them botanist Charles de l’Écluse, astronomers Tycho Brahe and Johannes Kepler, and painters Joris Hoefnagel and Roelandt Savery, were brought to the court (*4*). Prague became a major European cultural center with a characteristic flavor of science and the occult, which found its way into Arcimboldi’s work. He painted the Four Seasons twice during this time. Humanist Giovanni Fonteo composed verses, published in a separate booklet, expanding on the resemblances between the staying power of imperial rule and the cycles of nature.

Arcimboldi’s work has been ascribed to mannerism, the art of his times, known for its aesthetic quality, exaggeration, and emphasis on emotion. But his creative imagination moved in an entirely original direction. He turned elements from nature or everyday life into images of his own invention, transforming fruits, vegetables, flowers, animals, or books into enigmatic portraits. The parts were known and clearly understood, but the whole was new and elusive. For these elaborate illusionist tricks or “hieroglyphic wit,” poet and theologian Gregorio Comanini called Arcimboldi a “learned Egyptian” (*5*).

“For his long and conscientious service” (*3*), the painter was given permission to return to Milan, where he continued to work for Rudolph and completed his most famous works, Flora and the Nymph and Vertumnus―both of which he sent to Prague. He received the emperor’s highest orders for these paintings and died a year later.

Despite fame during his lifetime, Arcimboldi was soon forgotten to be rediscovered in our times. The century of Albert Einstein and Sigmund Freud took a closer look at the philosophy, symbolism, and sheer magic of his work. He was dubbed “the arch-father of surrealism” (*6*) for exerting influence on André Breton and Salvador Dalí. Pablo Picasso, who owned a copy of his Portrait of a Librarian (c. 1565), was inspired by it to paint the cubist Portrait of Daniel-Henry Kahnweiler (1910).

Arcimboldi’s Vertumnus, on this month’s cover, was the most famous work of art in Rudolph’s Prague. In this portrait, the emperor was shown as the Roman god of seasons, gardens, and plants, Vertumnus, who could change at will and was notorious for his disguises (Ovid Metamorphoses, Book XIV). The portrait was eulogized and explicated in a poem by Comanini: “If in looking you don’t admire/The ugliness that makes me handsome,/It’s that you don’t know how/ugliness surpasses/Every beauty.”

To deify Rudolph as lord of the seasons embodying the fruits of the world in a perpetual golden age, Arcimboldi rearranged nature’s bounty into a brand new ensemble. And by composing the emperor out of familiar parts, fruits and flowers, the painter reinvented him: “I vary from myself,/And thus, so varied, I am/One only, and from various things/With my varied countenance/I portray resemblances.”

“Look at the apple and the peach―/Round, red, and fresh―/That form both cheeks;/Turn your mind to my eyes―/One is a cherry,/The other a red mulberry,” exclaimed Vertumnus in Comanini’s poem, as if surprised by his own fantastic appearance. Composite creatures have fascinated throughout the ages. Hellenic mythology proposed Chimera, which appeared on pottery 2,500 years ago and was described by Homer in the Iliad (Book VI) as “a thing of immortal make, not human, lion-fronted and snake behind, a goat in the middle, and snorting out of breath of the terrible flame of bright fire.”

A tempting metaphor, Chimera has been adopted by many civilizations and, more recently, by various disciplines, among them genetics, molecular biology, and virology. Composites abound in nature. Those in the microbial world have gained notoriety in the face of emerging disease, one that Arcimboldi would have delighted in immortalizing. For this complex illusion, instead of fruits or flowers, he would have portrayed MRSA, avian influenza (H5N1), West Nile virus, *E. coli* O157:H7, and other hallmarks of emergence: ordinary parts rearranged in a new context. Its specter would have gone beyond astonishment to other common reactions evoked by the master’s unpredictable work: unease and foreboding.
